# Small Renal Masses: The Evolving Histologic, Imaging, and Genomic Landscapes

**DOI:** 10.3390/jcm12062361

**Published:** 2023-03-18

**Authors:** Julian Chavarriaga, Majed Al-Rumayyan, Ravi M. Kumar, Rui Bernardino, Rashid K. Sayyid

**Affiliations:** Division of Urology, Department of Surgery, Princess Margaret Cancer Centre, University of Toronto, 700 University Avenue, Toronto, ON M5G 1Z5, Canada

According to the American Cancer Society, it is currently estimated that approximately 81,800 new cases of kidney cancer will be diagnosed in the United States in 2023 [1]. This increased incidence is related, at least in part, to the increased utilization of axial abdominal imaging, with the subsequent incidental findings of small renal masses (SRMs). These lesions are characterized by an indolent natural history, with approximately 10–30% of SRMs being benign and those that are biopsy-proven malignant having a low metastatic potential (<2% metastatic at time of diagnosis) [2,3]. While percutaneous thermal ablation and extirpative procedures in the form of partial nephrectomies remain viable treatment options for SRMs, there has been an increased utilization of active surveillance (AS) in this setting. Longer-term follow-up of SRM AS cohorts have further characterized the natural history of such lesions. Recently, there has been emerging evidence that biopsy-based histopathologic characterization of SRMs may predict tumor growth patterns and future metastatic potentials [4].

*Renal Mass Biopsy Histology*

In 2020, histology-based outcomes of the largest AS cohort of patients with biopsy-proven malignant SRMs were reported. This cohort included 136 patients prospectively pooled from the Renal Cell Carcinoma Consortium of Canada and the Princess Margaret Cancer Centre [4]. Patients were inclusion eligible if they had cT1aN0M0 disease and had undergone a renal mass biopsy (RMB) for pathologic diagnosis. Serial imaging with computed tomography, magnetic resonance imaging (MRI), or ultrasound was performed at 3, 6, and 12 months, and annually thereafter. The median maximal tumor diameter at diagnosis was 2.3 cm (interquartile range [IQR]: 1.8–2.9 cm). At a median follow-up of 5.8 years (IQR: 3.4–7.5), the average growth rate in the maximal tumor diameter was 8% per year (0.17 cm in the 1st year; 0.19 cm/year over the first three years). Interestingly, growth patterns differed significantly by histology. In patients with papillary type 1 RCC, there were minimal changes in maximal tumor diameter or volume during follow-up (0.017 cm/year and −0.006 cm^3^/year, respectively). Conversely, the largest growth rates were observed in patients with clear cell renal cell carcinoma (ccRCC) at 0.28 cm/year. There was significant heterogeneity in the growth patterns of ccRCC SRMs (ranging from −0.03 to 1.0 cm/year), which hints at underlying genomic differences in these masses despite being of the same histologic subtype. Of the 134 patients, 6 (4%) developed metastatic disease, and all 6 had underlying ccRCC [4]. These findings reinforce the importance of RMB in this setting to enable prediction of SRM kinetics and metastatic potential, informing an individualized approach to SRM management.

*Imaging*

Imaging modalities have emerged as an adjunct to RMBs for the prediction of SRM histologic features. In a retrospective analysis of 103 patients with 109 SRMs resected between December 2011 and July 2015, pre-surgical renal MRI demonstrated sensitivities of 85% and 80% for the detection of clear cell and papillary RCC, respectively, with corresponding specificities of 76% and 94%, respectively. Inter-reader agreement was deemed moderate to substantial (clear cell RCC, κ = 0.58; papillary RCC, κ = 0.73), although this is likely to be inferior in non-tertiary care referral centers. While MRI appears as a promising tool for clear cell and papillary RCCs, this modality fared worse for predicting chromophobe histology, oncocytomas, and minimal fat angiomyolipomas, (sensitivity:14% to 67%; specificity: 97% to 99%), with fair to moderate inter-reader agreement (κ range = 0.23 to 0.43) [5].

Presented at the 2023 American Society of Clinical Oncology Genitourinary Symposium, the phase three ZIRCON study evaluated ^89^Zr-DFO-girentuximab for positron emission tomography computed tomography (PET/CT) imaging of ccRCC. Girentuximab is a monoclonal antibody that targets carbonic anhydrase IX, an enzyme highly expressed in ccRCC, and can thus aid differentiation between ccRCC and other histologic subtypes. In this open-label, multicenter trial, 300 patients with indeterminate renal masses (cT1: ≤7 cm) who were scheduled for a partial nephrectomy received a single dose of TLX250-CDx on Day 0 and subsequently underwent PET/CT imaging on day five (±2 days) prior to surgery. Using blinded central review via three independent readers, the mean sensitivity and specificity values for detecting ccRCC in the overall cohort were 86% and 87%, respectively. The corresponding positive and negative predictive values were 93% and 75%, respectively. A similar test performance was noted when the cohort was restricted to those with SRMs (i.e., cT1) [6]. When we consider the 14% non-diagnostic rate of RMBs for SRMs and that pathology from partial nephrectomies were used as the reference standard in the ZIRCON study, the results of this novel PET/CT-based imaging modality appear even more promising [7]. Furthermore, none of the ZIRCON study patients had any Girentuximab-related treatment-emergent grade ≥ 3 adverse events [6], whereas patients undergoing RMBs may experience hematomas and clinically significant pain in 5% and 1% of cases, respectively [7]. As such, this imaging modality may emerge as either an adjunct or replacement for RMBs, particularly in elderly, poor performance status patients on blood thinners and/or anti-platelet agents.

*Genomic Landscape*

The genomic profiling of patients with SRMs remains poorly understood. Studies exploring the mutational profiles of SRMs remain limited. This may be secondary to the decreased genomic mutational profile of smaller tumors, with the mutational burden increasing with tumor size [8], and due to limited tissue sampling from needle-core biopsies precluding molecular testing in this setting. Given the heterogeneity of SRM growth kinetics, even within the same histologic subtypes [4], a tumor-specific, genomic signature-informed approach will be key to optimizing patient selection for AS and minimizing risk of disease under- or over-treatment.

In a recent study of 1984 patients with SRMs, *BRCA1-*associated protein-1 (*BAP1*) loss was evaluated. This loss-of-function mutation is found in 15% of ccRCC lesions and is associated with worse survival outcomes [9]. *BAP1* status was found to be independently associated with time to metastasis for ccRCC (hazard ratio [HR]: 3.05; 95% CI: 1.30 to 7.15; *p* < 0.02). This association remained significant after adjusting for TNM (tumor-node-metastasis) stage and SSIGN (stage, size, grade, and necrosis) score in the multivariable analysis (HR: 3.58; 95% CI: 1.53 to 8.35; *p* < 0.003) [9].

A pooled analysis from The Cancer Genome Atlas program (TGCA), The International Cancer Genome Consortium (*n* = 31), University of Tokyo, and from NIH-NCI databases was performed for patients with ccRCC SRMs who underwent tumor genomic sequencing. A total of 203 tumors were analyzed, and mutations in *VHL* (62.6%), *PBRM1* (33.0%), *SETD2* (9.9%), *BAP1* (7.4%), *MTOR* (6.9%), and *KDM5C* (5.9%) were recorded. Notably, 3p subclonal driver mutations *KDM5C* (*p* = 0.005), *SETD2* (*p* = 0.023) and *BAP1* (*p* = 0.050) appeared to be significant predictors of increased recurrence and cancer-specific survival in the unadjusted analysis. However, this was only sustained for *KDM5C* after adjusting for confounders (*p* = 0.033) [10].

*Conclusions*

The current landscape of SRMs continues to evolve with the emergence of novel histology, imaging, and genomic-based modalities. Future growth/refinements of these modalities will be critical for the enhanced adoption of an individualized approach to the management of patients with SRMs.

## Data Availability

Not applicable.
